# Annual Meeting of the Belgian Society of Radiology (BSR): The Programme Engineered by the Young Radiologist Section (YRS)

**DOI:** 10.5334/jbr-btr.1451

**Published:** 2017-11-18

**Authors:** Ward Vander Mijnsbrugge

**Affiliations:** 1 University Hospitals Leuven, BE

As in previous years, the YRS has been largely involved in the preparation and the programming of the BSR’s meeting. This year, YRS has two topics running in parallel sessions: Neuroradiology and Pediatric Radiology.

The neuroradiology YRS session is entitled “The Brain Matters”, and will be moderated by Dr. Laurens Topff (ZOL, Genk) and Dr. Astrid Van Hoyweghen (UZA, Antwerp).

**Figure:**
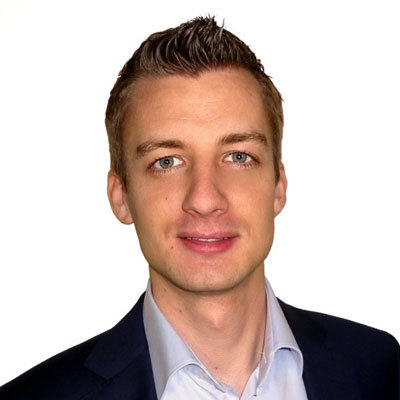
Laurens Topff

**Figure:**
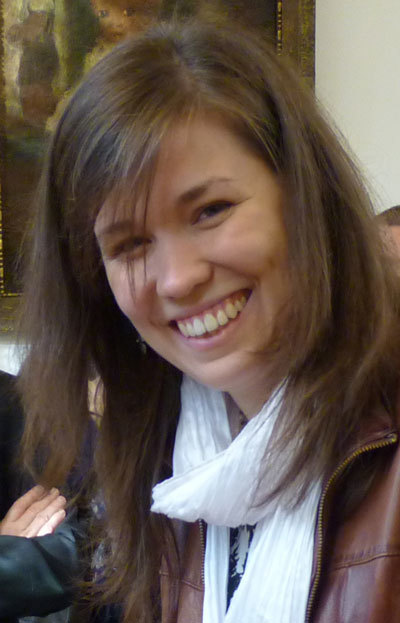
Astrid Van Hoyweghen

The first lecture will be given by Dr. Denis Brisbois, senior radiologist at the Centre Hospitalier chrétien of Liège and consultant at Hospital Erasme (Brussels), Centre Hospitalier régional (CHR) Liège and CHR Namur. He is a well-known neuro-interventional radiologist, having completed various fellowships in Europe (Geneva, Sutton, Reims) and the USA (Los Angeles). His lecture “How to Handle a Stroke Patient and What to Look For” is of great interest to every radiologist; he explains how the radiologist plays an important role in the management of stroke.

**Figure:**
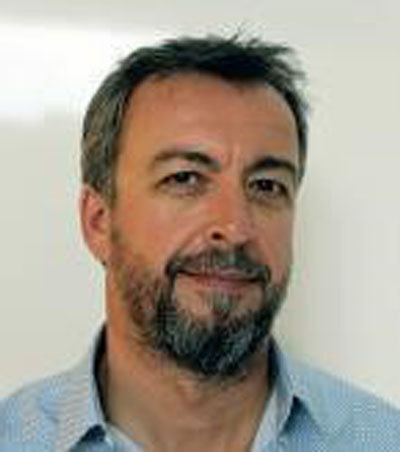
Denis Brisbois

Next, Prof. Dr. Alex Rovira, a pioneer in the diagnosis of demyelinating diseases, will enlighten us with his experience in the “Diagnosis of Multiple Sclerosis”. To cite a few of his accomplishments, Prof. Dr. Alex Rovira is head of the section of neuroradiology, director of the magnetic resonance unit at the Hospital Universitari Vall d’Hébron (Barcelona) and professor of radiology and neuroimmunology at the Autonomous University of Barcelona. Besides being the current President of the European Society of Neuroradiolgy, he is also a member of the executive committee of the Spanish Society of Radiology and co-chairman of MAGNIMS (European Multicentre Collaborative Research Network on MRI in MS).

**Figure:**
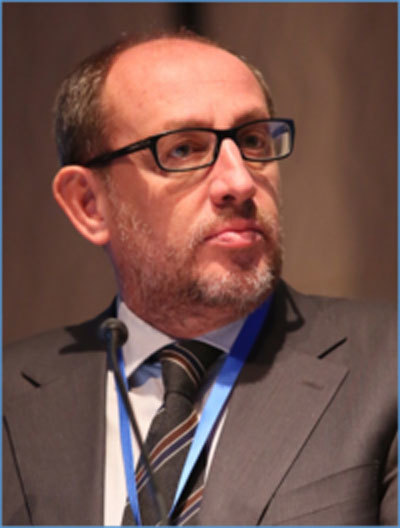
Alex Rovira

Amongst the sources of neurological damage, recreational drug use might be less recognized than vascular and demyelinating diseases. Nevertheless, neuroradiology is a cornerstone in the diagnosis of a variety of complications due to drug use. Accordingly, Dr. Luc van den Hauwe will give a lecture entitled “Neurocomplications of Recreational Drug Use”. Dr. Luc van den Hauwe is a neuroradiologist, working in AZ KLINA (Brasschaat) at the Antwerp University Hospital. He has been lecturer in many national and international scientific meetings (ECR, ESNR, ESMRMB) and courses such as the ESNR, the ESOR and the Erasmus Courses.

**Figure:**
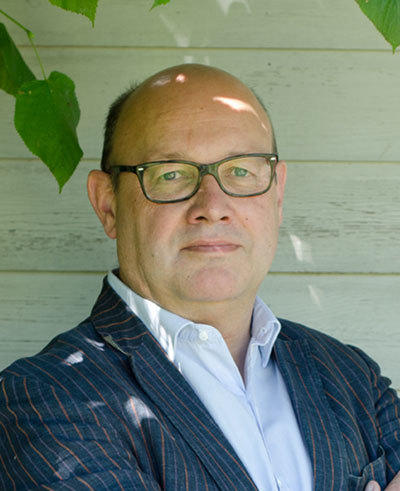
Luc van den Hauwe

The session ends with an interactive quiz-like presentation. This year Dr. Cedric Bohyn and Dr. Ward Vander Mijnsbrugge, both third-year radiology residents at the UZ Leuven, will tease your brain with tumour-like lesions in their presentation “A Brain Tumor, is it?” In between the voting, try not to forget to look closely at the images and the take-home messages.

**Figure:**
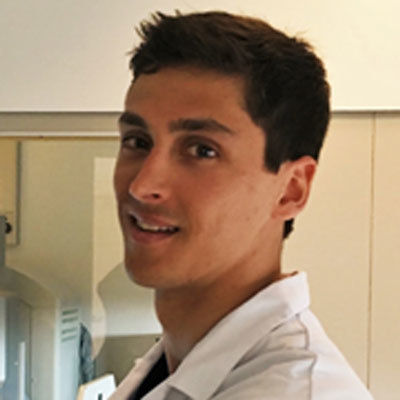
Cedric Bohyn

**Figure:**
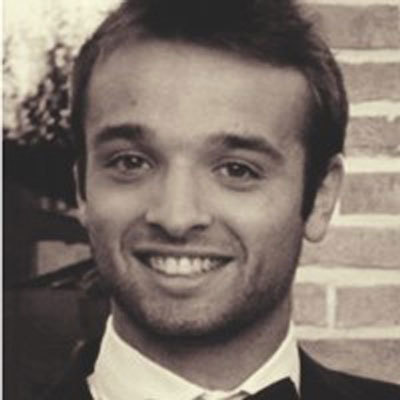
Ward Vander Mijnsbrugge

The pediatric radiology parallel YRS session is entitled: “Make Pediatric Radiology Great Again”. This session will be moderated by Dr. Laurent Van Camp (UZ Leuven) and Pierre-Antoine Poncelet (UCL).

**Figure:**
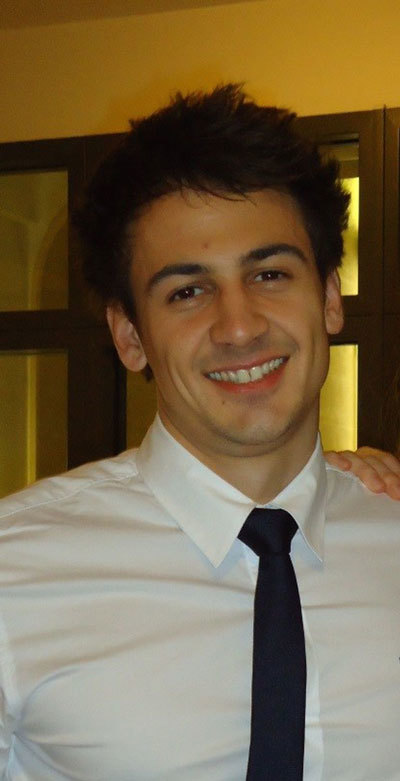
Laurent Van Camp

**Figure:**
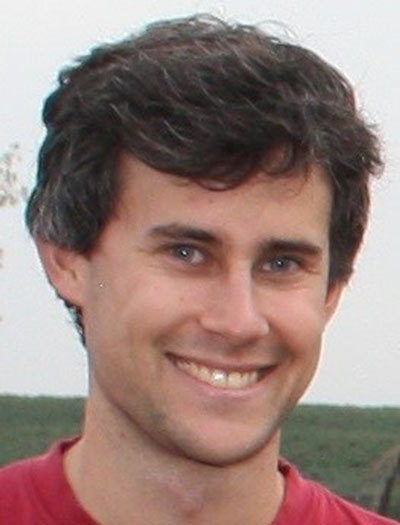
Pierre-Antoine Poncelet

The first lecture will be given by Dr. Michael Aertsen, aiming at providing an update on imaging in non-accidental trauma and emphasizing the guidelines of the European Society of Pediatric Radiology. Dr. Michael Aertsen is the youngest staff member in the pediatric radiology department at the UZ Leuven. He is preparing a PhD thesis on MRI of fetal brain development and is involved in several other projects, such as the impact of prenatal spina bifida repair, the improvement of prenatal CT protocols and post mortem imaging of fetuses. He is a member of the editorial board of the European Journal of Radiology for Prenatal Diagnosis.

**Figure:**
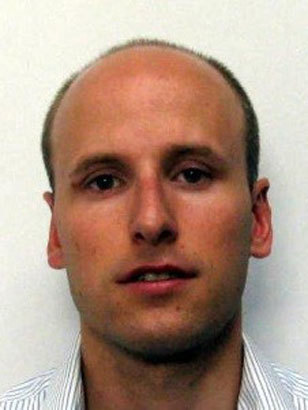
Michael Aertsen

During the next lecture, Prof. Dr. Michael Riccabona of the LKH in Graz (Austria), will highlight how powerful neonatal brain ultrasound is, for (pediatric) radiologists in the diagnosis and follow-up of various neonatal brain pathologies. Prof. Dr. Michael Riccabona is comprehensively presented in the editorial of Drs Desprechins and Breysem.

The third lecture will be given by Dr. Dana Dumitriu on “Uses and Abuses of Ultrasound in the Pediatric Emergency Room”. Dr. Dana Dumitriu is a staff member of the pediatric radiology department at Cliniques Universitaires Saint-Luc, UCL in Brussels. She has published several contributions in the fields of pediatric abdominal chest and bone imaging. She’s a strong advocate of maintaining ultrasound as a key instrument to pediatric radiologists and upholding the crucial role of the radiologist in the pediatric team.

**Figure:**
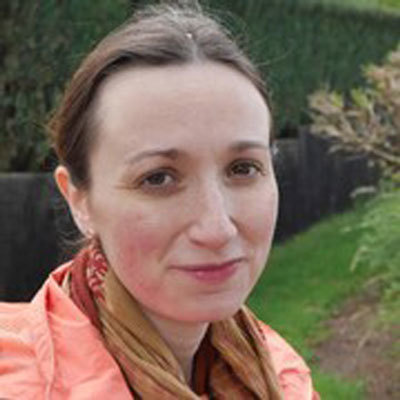
Dumitriu Dana

To end the session, there is an interactive quiz on pediatric oncology, presented by Dr. Nicolas De Vos and Dr. Anne-Sophie Vanhoenacker, respectively third-year radiology resident (UZ Gent) and second-year radiology resident (AZ Groenige Kortrijk – UZ Leuven). In between the voting, try not to forget to look closely at the images and the take-home messages.

**Figure:**
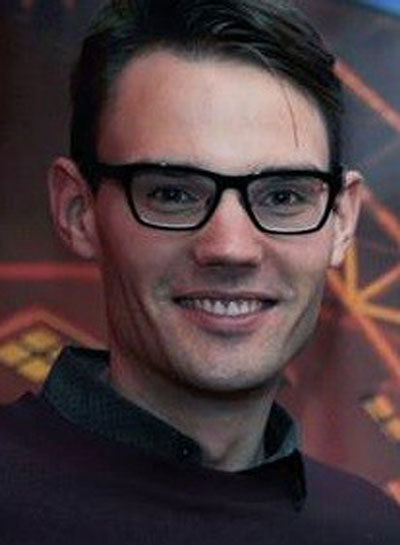
Nicolas De Vos

**Figure:**
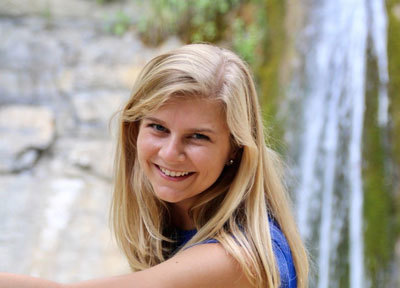
Anne-Sophie Vanhoenacker

The traditional Clash of the Titans will be moderated by the two chairs of the YRS section: Dr. Rita Lopes do Rosário (UCL, Brussels) and Dr. Naïm Jerjir (AZ Nikolaas, St-Niklaas.

**Figure:**
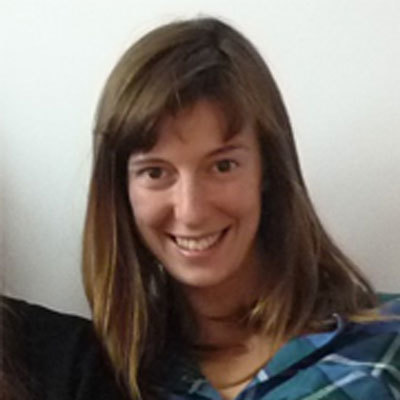
Rita Lopes do Rosário

**Figure:**
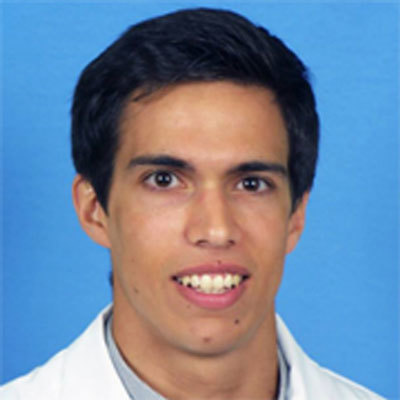
Naïm Jerjir

During the plenary Clash of the Titans session, three well-known pediatric and neuroradiologists will be faced with a difficult case – even for their standards. They will analyse the case, explaining how they deal with the differential diagnosis, giving tips and tricks to the audience. Our titans for pediatric radiology are Dr. Anne Smets, Prof. Dr. Freddy Avni and Dr. Dana Dumitriu. Our titans for neuroradiology are Prof. Dr. Françoise Dreyfus-Héran, Prof. Dr. Philippe Demaerel and Prof. Dr. Alex Rovira.

